# The advances in targeting CD47/SIRPα “do not eat me” axis and their ongoing challenges as an anticancer therapy

**DOI:** 10.18632/oncotarget.28607

**Published:** 2024-07-10

**Authors:** Maria Gracia-Hernandez, Manasa Suresh, Alejandro Villagra

**Keywords:** Immunotherapy, macrophages, CD47, HDACs

The growing understanding of the tumor immune microenvironment and its significance in shaping treatment outcomes has directed the latest advances in cancer therapy to boost antitumor immune responses. While most current immunotherapies focus on improving antitumor adaptive immunity, specifically T cells using immune checkpoint inhibitors (ICIs), there is an increasing interest in targeting innate immune components, such as macrophages. Macrophages represent a large percentage of immune cells in the tumor microenvironment (TME) of different cancer types. Tumor-associated macrophages (TAMs) promote tumor growth, metastasis, angiogenesis, and structural support. These characteristics have triggered intense interest in identifying novel immunotherapies to switch the M2-like (anti-inflammatory and pro-tumoral) macrophage population in the TME to the M1-like (pro-inflammatory and antitumoral) phenotype.

Besides their phenotypic plasticity, macrophages are also known for their phagocytic functions, and both pro-tumoral and anti-tumoral macrophages are phagocytic [1, 2]. However, the tumor immunology field frequently overlooked this crucial function of macrophages. Numerous phagocytosis checkpoints and “do not eat me” signals (i.e., CD47, CD24, MHC-I, PD-L1, STC-1, and GD2) have recently been recognized as potential therapeutic targets [3]. Recent reports have shown that multiple tumor types overexpress CD47 to evade phagocytosis upon interaction with the signal regulatory protein alpha (SIRPα) expressed by macrophages [4, 5]. Therefore, several therapeutic strategies to target this CD47/SIRPα axis have been explored recently, including monoclonal antibodies against CD47 or SIRPα [6], bispecific antibodies for CD47 and PD-L1 [7–9] or tumor-specific antigens [10, 11], recombinant SIRPα-Fc proteins to block CD47 or other peptide agonists [12–14], or tumor-selective CD47 blocking antibodies [15]. In addition to improving macrophage-mediated phagocytosis, macrophages that have phagocytosed cancer cells through CD47 blockade can prime CD8 T cells, but not CD4 T cells, to proliferate *in vitro* and *in vivo,* thus enhancing antitumor adaptive immunity [16]. Besides macrophages, anti-CD47 also triggers the cross-priming ability of DCs to activate T cells [17, 18].

The role of CD47 and the prospects of blocking its interaction with SIRPα have been widely studied in several preclinical models [19]. In a recent article, Al-Sudani et al. demonstrated that anti-CD47 antibodies have potent antitumor effects in both cell-derived and patient-derived xenograft models and enhance the macrophage-mediated phagocytosis of ovarian cancer cells *in vitro* via STING pathway [20]. However, the preclinical success of these approaches has not been replicated in the clinic [21]. Over the years, several strategies targeting or blocking the function of CD47 and SIRPα have been evaluated in clinical trials with marginal success rates. CD47 targeting agents face some challenges that need to be overcome to improve their antitumor efficacy (summarized in Table 1). The main limitation of current therapies targeting CD47 is the ubiquitous expression of CD47 in virtually every cell in the human body, therefore acting as an “antigen sink” and decreasing the specific targeting of the CD47 expressed on the tumors. In addition, the risk for anemia is of great concern, as aging red blood cells (RBCs) express higher levels of CD47 [22, 23]. Bouwstra et al. reviewed these limitations and addressed the potential solutions, including antibodies that bind specifically to CD47 on tumor cells and not in healthy cells or RBCs [21]. Blocking SIRPα instead of CD47 [21, 24] has been proposed as an alternative to avoid antigen sink, as SIRPα has a more restricted histological distribution than CD47 [25]. In addition, bispecific antibodies against CD47/PD-L1, CD47/CD38, and CD47/CD20, among others, successfully decrease RBC toxicity as they direct the antibodies to the tumor [7, 15, 26, 27]. In addition, the mode of administration is important to limit hematotoxicity. To illustrate, a priming dose followed by a higher maintenance or therapeutic dose has been shown to decrease hematotoxicity [22, 28]. In addition, local CD47 blockade can reduce toxicity and enhance its antitumor effects either as monotherapy or in combination with other immuno-oncology agents [29]. Besides toxicity concerns, optimizing the Fc domain of anti-CD47 antibodies can contribute to the development of more effective therapies. Osorio et al. demonstrated that local administration of anti-CD47 antibodies with engineered Fc domain has enhanced binding to the Fcγ receptors and hence promotes better infiltration of macrophages and antigen-specific T cells into the tumor and depletes regulatory T cells [30].

**Table 1:** Summary of challenges of CD47 targeting agents and potential solutions

Challenge	Potential solution
Ubiquitous CD47 expression acts as antigen sink	Local delivery of CD47 blocking agents. Tumor targeting agents (i.e., bispecific antibodies).
RBC toxicity	Target SIRPα instead of CD47. Develop agents that selectively target CD47 on tumor cells and not healthy cells such as RBCs. Administer priming dose followed by maintenance or therapeutic dose. Intratumoral delivery.
Limited antitumor activity	Combination therapies: immunotherapy such as other ICIs or tumor-targeting antibodies, chemotherapy, radiation therapy, PARP inhibitors, or epigenetic modifiers such as DNMT or HDAC inhibitors.

In a review article, Hua Yang and colleagues describe the current landscape and future prospects of CD47-based immunotherapy for hematological malignancies [31]. While CD47 blocking antibodies have shown success in hematological malignancies as monotherapy [32], their efficacy in monotherapy may be limited in solid tumors due to the heterogeneity and complexity of the TME [33]. Therefore, combination therapies are being evaluated to potentiate anti-CD47 immunotherapy in solid tumors. These combination therapies aim to: (1) enhance antibody-dependent cellular phagocytosis (ADCP) and antibody-dependent cellular cytotoxicity (ADCC) through Fc receptor engagement, (2) increase pro-phagocytic signals in the TME, such as calreticulin, (3) enhance T cell activation, and (4) induce repolarization of TAMs to a more antitumoral macrophage phenotype (M1-like) [34].

Therapies targeting the CD47/SIRPα axis are being evaluated in combination with chemotherapy agents, radiation therapy, PARP inhibitors [20], genotype-directed therapies [35], other tumor-targeting antibodies (i.e., CD20, VEGF, Her2), and epigenetic modifiers such as azacitidine [34]. Ye et al. summarized these preclinical studies where therapies targeting the CD47/SIRPα axis were used in combination with other agents, which showed an enhanced inhibition of tumor growth and/or improved survival compared to CD47/SIRPα axis disruption alone [34]. Although epigenetic modifiers such as azacitidine are being investigated in combination with CD47 blocking agents in clinical trials, there is a lack of preclinical studies assessing the efficacy of CD47 blocking agents in combination with histone deacetylase (HDAC) inhibitors. Recently, HDAC inhibitors were reported in the literature to enhance anti-CD47 immunotherapy. Our group recently published that HDAC6 inhibitors improve anti-CD47 immunotherapy by modulating the CD47/SIRPα axis on melanoma cells and macrophages, thus enhancing the macrophage-mediated phagocytosis of melanoma cells [36]. In this study, we administered the CD47 blocking antibody MIAP301 (IgG2a) intratumorally, in combination with the HDAC6 inhibitor Nexturastat A, to investigate the effects of the combination therapy on tumor growth and the immune cell populations in the TME. We also observed that the HDAC6 inhibitor Nexturastat A increased CD16/CD32 expression in naïve and M1-like macrophages (unpublished data), which can help explain how this HDAC6 inhibitor and CD47 work in combination. In addition, intratumoral delivery of anti-CD47 antibodies in combination with systemic administration of Nexturastat A significantly decreased SM1 melanoma growth by modulating the macrophage and natural killer cell populations in the TME. Other groups have also reported that other HDAC inhibitors modulate phagocytosis and the CD47/SIRPα axis. To illustrate, the Class I HDAC inhibitor Tacedinaline enhances phagocytosis and survival of MYC-driven medulloblastoma-bearing mice [37]. Laengle et al. reported that the HDAC inhibitors valproic acid (Class I) and vorinostat (Class I and II) enhance the expression of the activating antibody-binding receptor Fc-gamma receptor IIA on monocytes, induce immunogenic cell death, and decrease CD47 expression on tumor cells, thus enhancing phagocytosis [38]. However, most of these studies evaluate the role of different HDAC inhibitors in modulating phagocytosis instead of their antitumor efficacy in combination with CD47 blocking agents *in vivo*.

Overall, our understanding of this significant “do not eat me” pathway over the years has resulted in the development of novel therapies and strategies to boost innate antitumor activity. Considering the development of novel approaches to target the CD47/SIRPα axis while preventing the side effects described above, it would be interesting to test the synergy between these novel agents and HDAC inhibitors in preclinical models. Due to the role of HDACs in immunomodulation and tumor immunology [39, 40] and the diverse roles that the CD47/SIRPα axis plays in macrophage, DC, T cell, B cell, and NK cell function [41, 42], it is reasonable to foresee an increase in preclinical and clinical studies evaluating the antitumor efficacy and immunomodulatory properties of these combination therapies, as they could have broad roles in immune activation to combat tumors.
